# Tissue Distribution and Toxicological Risk Assessment of Mercury and Other Elements in Northern Populations of Wolverine (*Gulo gulo*)

**DOI:** 10.1007/s00244-024-01081-x

**Published:** 2024-08-03

**Authors:** John Chételat, Thomas S. Jung, Malik Awan, Steven Baryluk, William Harrower, Piia M. Kukka, Christine McClelland, Garth Mowat, Nicolas Pelletier, Christine Rodford, Raphaela Stimmelmayr

**Affiliations:** 1 Environment and Climate Change Canada, National Wildlife Research Centre, Ottawa, ON Canada; 2 Department of Environment, Government of Yukon, Whitehorse, YT Canada; 3 Department of Renewable Resources, University of Alberta, Edmonton, AB Canada; 4 Department of Environment, Government of Nunavut, Arviat, NU Canada; 5 Environment and Climate Change, Government of the Northwest Territories, Inuvik, NT Canada; 6 Department of Forest and Conservation Sciences, University of British Columbia, Vancouver, BC Canada; 7 Ministry of Forests, Government of British Columbia, Nelson, BC Canada; 8 Department of Earth, Environmental and Geographic Sciences, University of British Columbia, Kelowna, BC Canada; 9 Department of Geography and Environmental Studies, Carleton University, Ottawa, ON Canada; 10 Department of Wildlife Management, North Slope Borough, Utqiagvik, AK USA; 11 Institute of Arctic Biology, University of Alaska Fairbanks, Fairbanks, AK USA

## Abstract

Wolverines are facultative scavengers that feed near the top of terrestrial food chains. We characterized concentrations of mercury and other trace elements in tissues of wolverine from a broad geographic area, representing much of their contemporary distribution in northwestern North America. We obtained tissues from 504 wolverines, from which mercury was measured on muscle (n = 448), kidney (n = 222), liver (n = 148), hair (n = 130), and brain (n = 52). In addition, methylmercury, seven trace elements (arsenic, cadmium, chromium, cobalt, lead, nickel, selenium), and arsenic compounds were measured on a subset of samples. Concentrations of mercury and other trace elements varied between tissues and were generally highest in kidney compared to brain, liver and muscle. Mercury was predominately as methylmercury in brain and muscle, but largely as inorganic mercury in liver and kidney. Mercury concentrations of hair were moderately correlated with those of internal tissues (Pearson r = 0.51–0.75, *p* ≤ 0.004), making hair a good non-lethal indicator of broad spatial or temporal differences in mercury exposure to wolverine. Arsenobetaine was the dominant arsenic compound identified in tissues, and arsenite, arsenocholine and dimethylarsinic acid were also detected. A preliminary risk assessment suggested the cadmium, lead, mercury, and selenium concentrations in our sample of wolverines were not likely to pose a risk of overt toxicological effects. This study generated a comprehensive dataset on mercury and other trace elements in wolverine, which will support future contaminants study of this northern terrestrial carnivore.

**Supplementary Information:**

The online version contains supplementary material available at 10.1007/s00244-024-01081-x.

The wolverine *Gulo gulo* (Linnaeus, 1758) is the largest terrestrial species in the mustelid family and is widely distributed across remote high latitude regions of North America, Europe, and Asia (Glass et al. 2022; Copeland et al. 2010). In North America, its distribution extends southward into boreal and montane ecozones in the west but wolverines have been largely extirpated from central and eastern regions of the continent (Fisher et al. 2022). Wolverines are facultative scavengers and opportunistic predators that consume a variety of large and small prey, ranging from large ungulates like moose *Alces americanus* to smaller mammals such as snowshoe hare *Lepus americanus* and marmots *Marmota* spp. (Fisher et al. 2022; Inman et al. 2012a; Mattisson et al. 2016; Lofroth et al. 2007; Dorendorf et al. 2018). At higher latitudes in Canada and Alaska, caribou *Rangifer tarandus* are a main food source that they obtain by hunting or scavenging (Fisher et al. 2022; GB-W-BMFWG 2022). Wolverines living in coastal regions also scavenge the remains of long-lived marine mammals (Glass et al. 2022). Their relatively large home range (Inman et al. 2012b; Dawson et al. 2010), high trophic position, diverse and seasonally variable diet, and broad distribution make wolverines an ecologically relevant sentinel species in high latitude terrestrial food webs. They also are a bio-culturally important species to Indigenous peoples in northern socio-ecological systems (Glass et al. 2022; GB-W-BMFWG 2022).

Terrestrial food webs in remote regions are exposed to metals through long-range atmospheric transport (Gamberg et al. 2005b). Mercury is transported long distances in gaseous form via air currents and deposits in remote regions far from anthropogenic emission sources. Inorganic mercury from the atmosphere is deposited on terrestrial and aquatic environments, where it is transformed into methylmercury (MeHg) via bacterial metabolism, bioaccumulates in food webs, and biomagnifies to higher concentrations in top predator animals (Shore et al. 2011). The environmental fate of mercury is complex, due to a multitude of transport, biogeochemical, ecological, and biological processes, and considerable effort has focused on characterizing those processes due to risk of toxicological effects on wildlife and humans at elevated levels of MeHg exposure (AMAP 2021). Other trace metals such as cadmium and lead are also transported via the atmosphere bound to ultra-fine particles and deposited on remote environments (e.g., Wiklund et al. 2020), where bioaccumulation may occur. However, unlike MeHg, most metals and metalloids do not biomagnify in food webs (Sun et al. 2020).

Local sources of metals can also contribute to metal bioaccumulation in terrestrial food webs through releases from natural processes (e.g., geological weathering, wildfire emissions) and human activities (e.g., industrial and urban developments) (Gamberg et al. 2005b; Kalisińska 2019). Wildfires in high-latitude regions, for example, release large amounts of mercury into the atmosphere through combustion of soils and plant biomass (Kumar and Wu 2019). Local geological sources of metals can also be significant, such as for cadmium in tissues of Arctic wolves *Canis lupus* and moose, which were found to be higher in the Yukon compared to other northern regions (Gamberg and Braune 1999; Gamberg et al. 2005a). Likewise, isotopic evidence suggested that regional fossil fuel emissions contributed to lead bioaccumulation in wildlife of northwestern Canada (Chételat et al. 2022). Elevated arsenic in hares near Yellowknife was associated with proximity to legacy pollution from gold mining (Amuno et al. 2018). Thus, both local releases and long-range transport can contribute to metal bioaccumulation in terrestrial wildlife.

Few data are available on contaminant burdens in wolverines. To our knowledge, only two studies have been published on metals concentrations in wolverines in Canada, specifically from British Columbia (Harding 2004) and Nunavut (Hoekstra et al. 2003a), and these were based on a small number of animals (n = 9–11). Hoekstra et al. (2003b) also reported concentrations of persistent organic pollutants in the same animals. In contrast, other species in the mustelid family, notably river otter *Lontra canadensis*, mink *Neovison vison* and marten *Martes americana* have been extensively studied for mercury and other metal exposures, including investigations of captive animals to evaluate toxicological effects (Wobeser et al. 1976; Wren et al. 1986; Langlois and Langis 1995; Harding et al. 1998; Basu et al. 2010; Klenavic et al. 2008; Evans et al. 2016; Eccles et al. 2017; Witt et al. 2020; Thomas et al. 2021). For instance, elevated mercury concentrations, associated with a risk of toxicological effects, have been observed for some river otter and mink in the wild. This is likely a result of their elevated trophic position and piscivorous diet, coupled with greater MeHg bioavailability in the aquatic ecosystems they inhabited (Basu et al. 2005; Yates et al. 2005).

Diet is the dominant exposure pathway for metal bioaccumulation in wildlife that inhabit areas far from anthropogenic point sources (Smith et al. 2007; Kalisińska 2019). Following ingestion, bioavailable metals in the gut cross the intestinal wall, enter the bloodstream and circulate to other tissues in the body (Scheuhammer 1987). Though target organs can differ between elements, metals commonly accumulate in the liver and kidney where detoxification processes occur (Kalisińska 2019). Excretion from the body occurs via urine and feces, maternal transfer, and sequestration in inert keratinous tissues (e.g., hair, claws) (Kalisińska 2019; Chételat et al. 2020). The physiological processes involving absorption, accumulation, elimination and toxicity differ among trace elements as well as between chemical forms (speciation) of an element. For example, many mammalian species have the capacity to methylate inorganic arsenic that is absorbed from diet, which results in detoxification and more rapid excretion of the arsenic (Vahter 1999). Inorganic mercury is less readily assimilated and more rapidly eliminated from the body than MeHg (Bradley et al. 2017). Inorganic forms of arsenic and selenium are more toxic than organic forms (Pilarczyk et al. 2019; Binkowski 2019), while MeHg is more toxic than inorganic mercury (Shore et al. 2011). The bioaccumulation of metals and metalloids involves complex physiological processes that vary among vertebrate species (Omata et al. 1986; Vahter 1999; Chételat et al. 2020). Thus, measurement of element concentrations (and speciation when possible) in multiple tissues allows for a more comprehensive assessment of exposure to wildlife.

The overarching objective of this study was to characterize trace element concentrations in multiple tissues of wolverine from a broad geographic area of western North America. A complementary investigation of the same wolverines, reported in Peraza et al. (2023), focused on an evaluation of environmental processes and biological factors controlling spatial patterns of mercury bioaccumulation. Here, we measured mercury in 1000 tissue samples, and additionally five other metals (cadmium, chromium, cobalt, lead, nickel) and two metalloids (arsenic, and selenium) were measured on a subset of 100 samples. The selected trace elements were investigated because they are listed as toxic substances by the Government of Canada (GOC 2021). Total concentrations were measured for most elements, though the chemical speciation of mercury and arsenic were examined on a subset of samples. Trace element concentrations were measured in various tissues (i.e. brain, kidney, liver, and muscle) to evaluate their internal distribution. Hair was also included to evaluate non-lethal sampling of a keratinous tissue as a biomarker for mercury exposure. A risk assessment was performed for potential toxicological effects of the bioaccumulated metals and metalloids.

## Materials and Methods

### Sample Collection

**Fig. 1:**
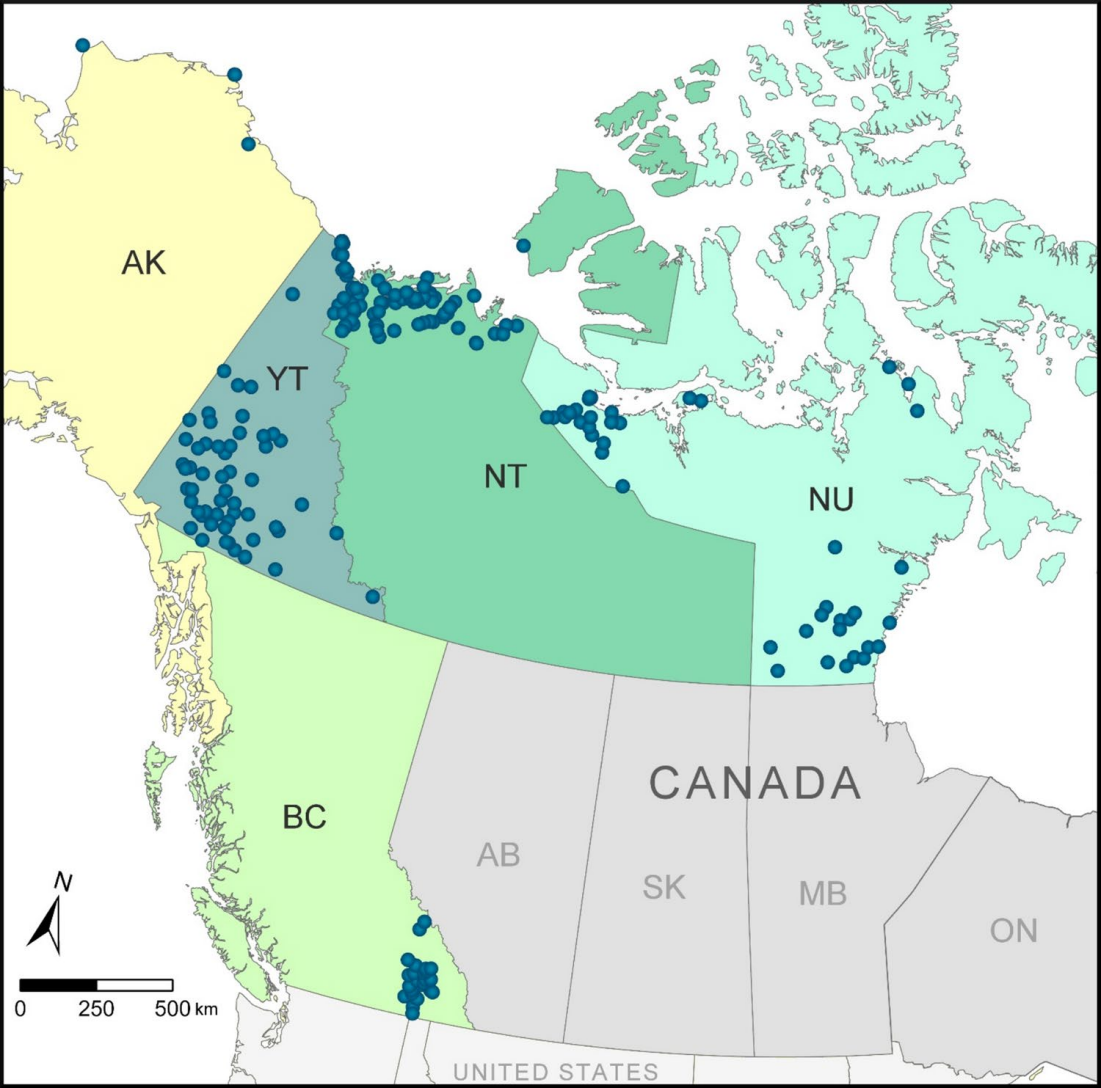
Map showing the approximate locations of wolverine sample collections in western North America. Wolverines were sampled from British Columbia (BC), the Yukon (YT), the Northwest Territories (NT), and Nunavut (NU) in Canada, and from Alaska (AK) in the United States of America. Map sources: Esri Canada, Natural Earth Vector, U.S. Department of Commerce, Census Bureau; U.S. Department of Commerce, National Oceanic and Atmospheric Administration, National Ocean Service, National Geodetic Survey

Wolverine tissues were obtained between 2005 and 2018 through carcass collection programs in the Yukon, Northwest Territories, and Nunavut (Canada) (e.g., Jung et al. 2020; Kukka et al. 2017) as well as the North Slope Borough of Alaska (United States of America). Hair was also obtained from wolverines through hair snag monitoring in the interior of British Columbia (Canada) (Kortello et al. 2024). The study area included wolverines sampled in five jurisdictions and covered more than 1,600,000 km^2^, representing a large portion of the species range in western North America (Fig. 1).

Carcasses submitted by Indigenous or licenced trappers were dissected to remove the brain, liver, kidney, muscle (hind or masseter), and hair, although not all tissues were obtained from each animal. Sample collection included tissues from 504 wolverines, from which one or more of the following tissue types were available: muscle (n = 448), kidney (n = 222), liver (n = 148), hair (n = 130), and brain (n = 52). We focused on wolverines from the Yukon for some chemical analyses (where only a subset of samples could be analyzed) because complete tissue sets (brain, liver, kidney, muscle) were available from individual animals collected in that jurisdiction. Ancillary collection and biological information for each animal was obtained where possible, specifically collection date, location coordinates, and sex. Across all study regions, more males were collected than females, in approximately a 2:1 ratio, which is characteristic of most regional wolverine harvest patterns (Kukka et al. 2017). Age was estimated for a subset of animals based on an analysis of cementum layers of canine teeth. The wolverines in our study (for which data are available) ranged in age from < 1 to 16 years, with a median age of 3 years. Details on the sample set, specifically profiles of age class, sex, and years of collection by study region, are provided in Table S1 of the Supplementary Information.

### Chemical Analysis

Preparation of wolverine tissues was performed in a trace metal clean laboratory at the National Wildlife Research Centre (NWRC; Ottawa, Ontario, Canada). Prior to chemical analysis, brain, kidney, liver, and muscle were freeze-dried and homogenized. Kidney and liver were homogenized with an Omni Mixer before drying while brain and muscle were manually homogenized in their containers before and after drying, respectively. Hair was washed with a 2:1 chloroform:methanol solution to remove dirt and oils and left for 24 h to air dry in a fume hood (Blight et al. 2015).

Total mercury (THg) was measured on all wolverine samples at NWRC using a Direct Mercury Analyzer (DMA-80, Milestone Inc., Italy) by combustion, gold trapping, and detection with cold vapor atomic absorption spectrophotometry. Methylmercury was measured at NWRC in brain, kidney, liver and muscle of 25 wolverines from the Yukon. For each animal, all four tissues were analyzed allowing for a cross-tissue comparison of the proportion of THg as MeHg. Methylmercury was extracted from samples in nitric acid for 16 h at 60 °C, ethylated with sodium tetraethylborate, concentrated on a Tenax trap, separated by gas chromatography, and detected by cold vapor atomic fluorescence spectrometry with a Tekran 2700 (Tekran Instruments Corporation, Scarborough, Ontario, Canada). A suite of metals and metalloids was measured in brain, kidney, liver and muscle of the same 25 wolverine at a commercial laboratory (RPC; Fredericton, New Brunswick, Canada). Total concentrations of arsenic, cadmium, chromium, cobalt, lead, nickel, and selenium were measured by inductively coupled plasma mass spectrometry (ICP-MS) following microwave-assisted sample digestion in nitric acid. Arsenic speciation was characterized for brain, liver and muscle of three wolverine from the Yukon at a commercial laboratory (ALS; Burnaby, British Columbia, Canada). Homogenized wet samples were analyzed for arsenate, arsenite, arsenocholine, arsenobetaine, dimethylarsinic acid, and monomethylarsonic acid following enzyme digestion and detection by anion exchange, high performance liquid chromatography (HPLC), and Collision/Reaction Cell ICP-MS. Concentrations are reported on a dry weight (dw) basis except for hair, which is reported as fresh weight (fw). Arsenic speciation results are reported as a proportion of total arsenic concentration in the sample.

### Quality Control/Quality Assurance

For THg measurements, a blank, analytical duplicate, and certified reference material (CRM) were analyzed for every 10 samples. Blanks were typically < 0.07 ng and results were above the analytical reporting limit of 0.2 ng. The relative percent difference (RPD) of duplicates was on average 6 ± 7% (n = 144, ± standard deviation). Accuracy of the instrument was evaluated by mean recovery (± standard deviation) of the following CRMs: Tuna Fish Flesh Homogenate IAEA-436 (99 ± 8%, n = 47), TORT-3 Lobster Hepatopancreas (102 ± 5%, n = 157), DOLT-5 Dogfish Liver (92 ± 6%, n = 17), and NIST-2976 Mussel Tissue (102 ± 5%, n = 51).

For MeHg measurements, concentrations were blank-corrected, and results were above the analytical detection limit of 10 ng/g. The RPD of duplicates was on average 3 ± 3% (n = 16). Accuracy of the instrument was evaluated by mean recovery (± standard deviation) of the following CRMs: Tuna Fish Flesh Homogenate IAEA-436 (106 ± 4%, n = 8), TORT-3 Lobster Hepatopancreas (88 ± 4%, n = 8), DORM-4 Fish Protein (87 ± 2%, n = 8), and NIST-2976 Mussel Tissue (93 ± 7%, n = 17).

For metal and metalloid measurements by ICP-MS, detailed presentation of data for analytical duplicates, detection limits, and CRM recoveries are presented for each element in the Supplementary Information (Table S2). Blanks (n = 7) were below analytical detection for arsenic, cadmium, cobalt, and nickel. Selenium was detected in one blank (0.012 µg/g) at a level that was < 5% of the minimum concentration in samples. Chromium was detected in three blanks (0.08–0.12 µg/g) at levels > 10% of some samples. All samples had concentrations above analytical detection for cobalt, nickel and selenium, while 1–25% of samples were below detection for arsenic, cadmium, chromium and lead. The average RPD of duplicates was 1–11% (n = 8–12) except for nickel and chromium, which had average RPDs of 22% and 39%, respectively. Accuracy of the instrument was evaluated by recovery of DOLT-5 Dogfish Liver (n = 7), DORM-4 Fish Protein (n = 8), and NIST-2976 Mussel Tissue (n = 3), and element recoveries averaged 94–107% with a range of 80–117%. Overall, chromium results for sample duplicates showed lower precision (average RPD of 37%), possibly due to minor method contamination and/or values close to the detection limit, but the CRMs indicated acceptable accuracy (recovery of 80–112%) and precision (< 5%) for that element (Table S2).

For arsenic speciation, detailed presentation of data for analytical duplicates, detection limits, and CRM recoveries are presented for each compound in the Supplementary Information (Table S3). No target arsenic species were detected in the method blanks (n = 2). Some target arsenic species were not detected in most samples. Recoveries from a reference material (n = 2) and laboratory control sample (n = 2) used by the commercial lab were within acceptable limits (80–120%).

### Data Analysis

The dataset of mercury and trace element concentrations in wolverine tissues is publicly available on the Government of Canada Open Data Portal at https://search.open.canada.ca/data/. Note the same muscle THg data were also reported in the supplemental information by Peraza et al. (2023).

For this study, the detection limit was used when element concentrations were below analytical detection. A total of five exceptionally high lead results were removed as outliers due to likely tissue contamination from lead ammunition used by hunters. This interpretation is supported by lead stable isotope analysis of those samples as reported in Chételat et al. (2022). Raw data were log-transformed or square-root transformed to meet assumptions of statistical testing by Pearson correlation or simple linear regression. Kruskal–Wallis one way analysis of variance on ranks (with Dunn’s post-hoc comparisons) was used to test for differences in the concentrations of metals and metalloids among tissues.

## Results and Discussion

### Mercury and Methylmercury

**Fig. 2:**
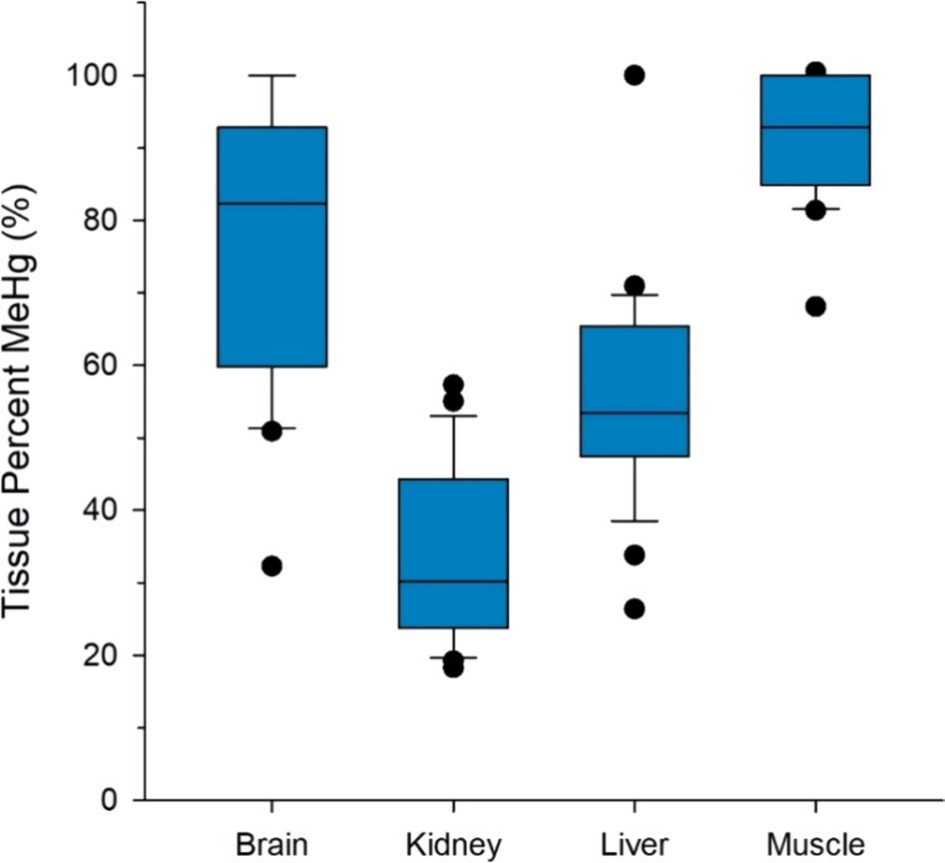
Box plots of the proportion of THg as MeHg in tissues of 25 wolverines from the Yukon, Canada. Error bars represent the upper 95% and lower 5% of observations. Dots are outliers

Total mercury concentrations of wolverine tissues varied by three orders of magnitude from 0.01 to 16.10 µg/g dw (Table 1). Mean and maximum concentrations differed between tissues, with the highest values in the kidney followed by hair, muscle, liver, and brain. The variation in THg concentrations reflects tissue-specific bioaccumulation processes as well as biological factors (i.e. sex, age), differences in dietary exposure to mercury, and timing of sampling. For the subset of wolverines examined from the Yukon, the proportion of THg as MeHg also varied between tissues (Fig. 2). Muscle and brain contained mercury that was predominately as MeHg (on average 94% and 77% of THg, respectively) while liver and kidney contained considerably less MeHg (on average 57% and 34% of THg, respectively).

**Table 1:** Mean and standard deviation, range (in parentheses) and sample sizes of THg concentrations in wolverine tissues from five regions in western North America

Region	Brain THg (µg/g dw)	Hair THg (µg/g fw)	Kidney THg (µg/g dw)	Liver THg (µg/g dw)	Muscle THg (µg/g dw)
Alaska			1.24 ± 0.56 (0.40–2.51) *n* = 16	0.29 ± 0.15 (0.13–0.68) *n* = 17	0.33 ± 0.21 (0.15—0.70) *n* = 13
British Columbia		0.28 ± 0.29 (0.04–1.40) *n* = 22			
Northwest Territories	0.25 ± 0.30 (0.02–1.46) *n* = 27	2.81 ± 2.21 (0.27–11.13) *n* = 33	3.17 ± 3.04 (0.16–16.10) *n* = 78	0.65 ± 0.73 (0.04–1.98) *n* = 10	0.89 ± 0.94 (0.05–5.72) *n* = 71
Nunavut		2.86 ± 2.47 (0.70–10.76) *n* = 23			0.72 ± 0.50 (0.13–2.03) *n* = 44
Yukon	0.11 ± 0.17 (0.02–0.71) *n* = 25	0.47 ± 0.46 (0.07–2.06) *n* = 52	0.90 ± 1.27 (0.06–6.85) *n* = 128	0.18 ± 0.26 (0.01–2.07) *n* = 121	0.22 ± 0.26 (0.01–1.78) *n* = 320
All regions	0.18 ± 0.26 (0.02–1.46) *n* = 52	1.46 ± 1.95 (0.04–11.13) *n* = 130	1.72 ± 2.31 (0.06–16.10) *n* = 222	0.22 ± 0.32 (0.01–2.07) *n* = 148	0.38 ± 0.53 (0.01–5.72) *n* = 448

The differences in percent MeHg among tissues reflect demethylation and elimination processes. For mammals, the liver is considered a primary organ for MeHg demethylation to inorganic mercury, although demethylation can also occur in kidneys, the gastro-intestinal tract and the brain (Chételat et al. 2020). Low percent MeHg is considered an indicator of active demethylation in the organ (Vahter et al. 1995; Eagles-Smith et al. 2009), and other lines of evidence for active demethylation include the presence of mercury-selenide nanoparticles (Gajdosechova et al. 2016) and mercury stable isotope tracing (Li et al. 2022; Evans et al. 2016). In a dosing experiment, mink fed a diet containing isotope-specific MeHg showed conversion of MeHg to inorganic mercury in both liver and kidney (Evans et al. 2016). Kidney is also an elimination route for inorganic mercury via urine (Farris et al. 1993), which could contribute to a low percent of MeHg in that organ. There appears to be considerable variation among vertebrate species in their capacity for physiological depuration of MeHg (Chételat et al. 2020). For example, there is evidence for MeHg demethylation in the brain of several species of non-human primates (Vahter et al. 1995) and long-finned pilot whales *Globicephala melas* (Gajdosechova et al. 2016) but not for polar bear *Ursus maritimus* (Krey et al. 2015) and mink (Evans et al. 2016). The proportion of THg as MeHg in the brain of wolverines (32–100%) suggests demethylation can also occur in that organ for this species, though further study is needed for a more definitive assessment.

**Fig. 3:**
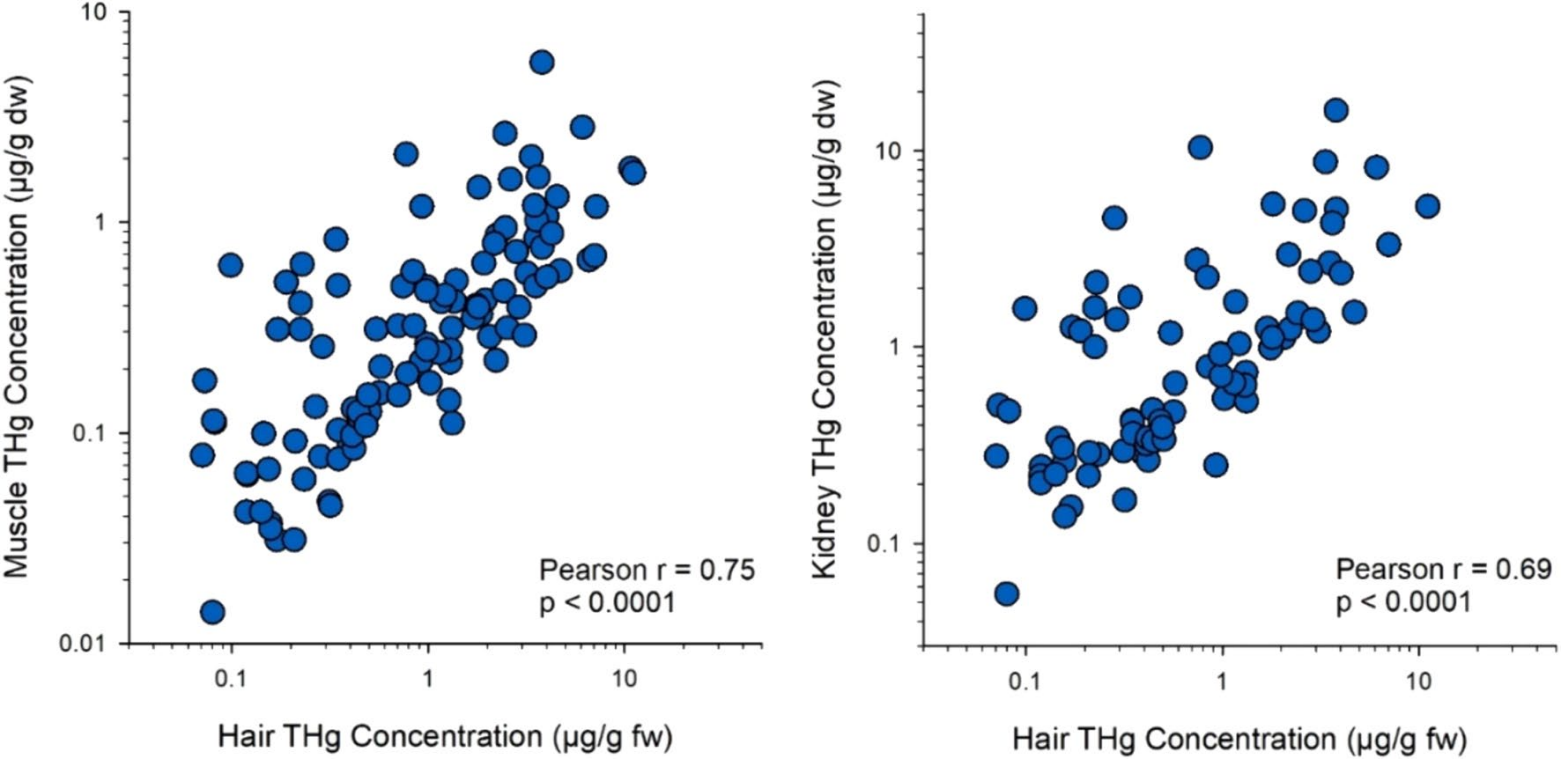
Correlations of THg concentrations in hair with muscle (left panel) and kidney (right panel) of wolverines. The data are presented on a logarithmic scale

The tissue types examined in our study showed good comparability for use as indicators of mercury exposure to wolverines (Table 2). There were strong positive correlations of THg concentration between internal tissues (Pearson coefficients = 0.89–0.96, *p* < 0.0001). Hair THg was also positively correlated with the THg of internal tissues, though the associations were weaker (Pearson coefficients = 0.51–0.75, *p* ≤ 0.004) (Fig. 3). Thus, the positive correlations between tissue types indicates that animals with higher mercury exposure bioaccumulated more THg in all the tissues examined.

**Table 2:** Pearson coefficients and sample sizes for correlations between tissues of log-transformed THg concentrations in wolverine

	Brain	Hair	Kidney	Liver
Hair	0.72 (*n* = 14)			
Kidney	0.96 (*n* = 37)	0.69 (*n* = 79)		
Liver	0.92 (*n* = 25)	0.51 (*n* = 54)	0.89 (*n* = 141)	
Muscle	0.96 (*n* = 47)	0.75 (*n* = 107)	0.93 (*n* = 195)	0.94 (*n* = 143)

All correlation coefficients were highly significant (*p* < 0.0001) except for the correlation between brain and hair THg concentration (*p* = 0.004)

The positive correlations between mercury concentrations in hair and internal tissues indicates hair is a useful non-lethal biomarker to evaluate large gradients of mercury exposure in wolverine. However, caution is warranted in interpreting small differences in hair THg concentrations because of the influence of other factors. Studies of other mammalian species have similarly reported variability with hair as a proxy of internal THg such as for polar bear (Bechshoft et al. 2019), Arctic fox *Vulpes lagopus* (Treu et al. 2018) and several species of insectivorous bats (Chételat et al. 2018; Yates et al. 2014). Lower correlations between THg concentrations of hair and internal organs could be due to spatial variation of MeHg uptake within the fur coat (Eccles et al. 2019), timing of hair collection and molting cycle, or a mismatch in timing between hair growth and more recent dietary exposure (Peterson et al. 2016).

Empirical models relating THg concentrations between different tissues of two mustelid species, mink and river otter, were previously reported by Eccles et al. (2017). These conversion factors, based on linear regression slopes, are a predictive tool that allows for comparisons of the internal distribution of mercury among species.. Here we report new empirical models for wolverine (Supplemental Table S4) and compare among-species variation in the distribution of THg between internal tissues of wolverine, otter, and mink. All three species showed a similar distribution of THg in the brain relative to muscle (Supplemental Table S5). In other words, all three species had similar conversion factors between THg in muscle and brain. In contrast, THg concentrations in the kidney were higher (relative to muscle) in wolverines than in river otters and especially mink. Conversion factors between liver THg and muscle were highest in river otter, followed by mink then wolverine (Supplemental Table S5). These empirical models of the internal distribution of THg in wolverines may be useful for future assessment of mercury exposure to wildlife in high-latitude regions (e.g., Dietz et al. 2022).

Our observations are consistent with previous studies showing species-specific differences in the internal processing of MeHg (Petersson et al. 1989; Omata et al. 1986). In this case, the kidneys were a more active organ for MeHg depuration in wolverine compared to published data for the liver of river otter and mink (Eccles et al. 2017). It has been noted that terrestrial mammals tend to have higher concentrations of mercury in the kidney than liver, while aquatic (piscivorous) mammals have higher concentrations in their liver (Gamberg et al. 2015; Kalisińska 2019; Dietz et al. 2013). This pattern could explain the differences between the three mustelid species; however, it does not always hold true such as in the case of Arctic fox, which had higher THg concentrations in liver than kidney (Treu et al. 2018). Differences in mercury distribution between tissues of terrestrial and aquatic-feeding mammals may be due to diet (Kalisińska 2019) and the higher proportion of inorganic mercury in plants and primary consumers in terrestrial food webs (Gamberg et al. 2015).

Peraza et al. (2023) conducted a separate analysis of muscle THg data reported in the current paper, examining environmental and biological influences on mercury bioaccumulation in wolverines. They found muscle THg concentrations of wolverines differed geographically, with the highest concentrations in animals near the Arctic Ocean coast. Diet (assessed with nitrogen stable isotope ratios) and landscape variables (soil organic carbon, percent cover of wet area, percent cover of perennial snow-ice, distance to coast) were significant explanatory variables of mercury in wolverines. Therefore, the wide variation in THg concentrations of wolverines reported here was largely due to differences in diet and landscape characteristics across our immense sampling area. A diet composed partially of food items of marine origin (i.e. cetaceans, pinnipeds, seabirds) may have enhanced mercury bioaccumulation in some wolverines, a phenomenon that has also been reported for Arctic fox (Bocharova et al. 2013; Hallanger et al. 2019) and gray wolves *Canis lupus* (McGrew et al. 2014). Biological factors can also influence mercury bioaccumulation in wildlife. Peraza et al. (2023) reported no difference in muscle THg concentration between male and female wolverines but higher concentrations in adults than juveniles. The age effect, however, was relatively minor compared to spatial differences related to diet and landscape features.

### Other Trace Elements

**Fig. 4:**
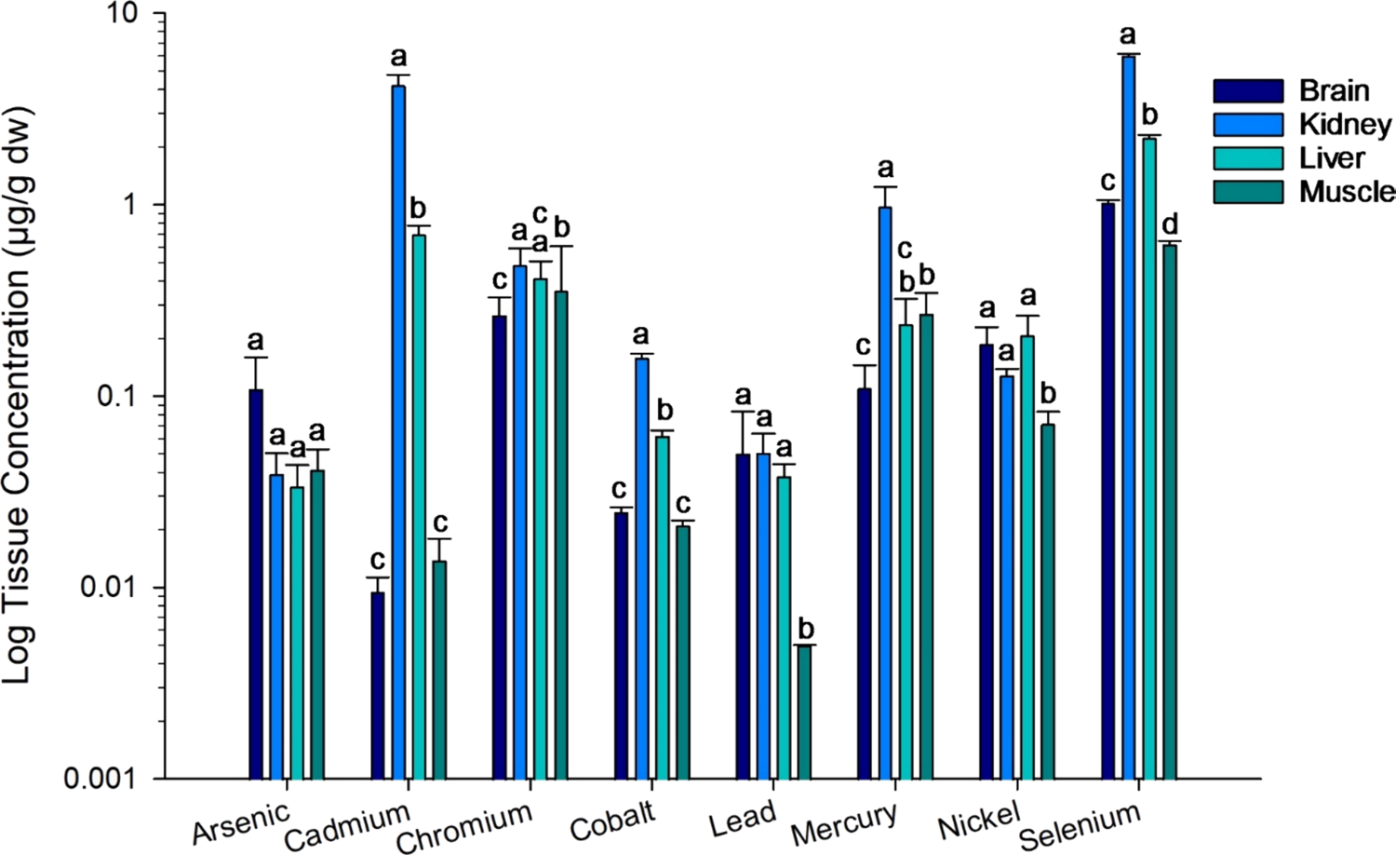
Means (± standard error) of trace element concentrations in brain, kidney, liver, and muscle of 25 wolverines from the Yukon, Canada. Concentrations are presented on a logarithmic scale. Letters above the bars indicate results of Dunn’s post-hoc comparisons of tissue differences by individual element. Note lead concentrations in muscle were all below analytical detection and the detection limit value is presented here

Trace elements in 25 wolverines from the Yukon ranged three orders of magnitude and differed by element and tissue type (Fig. 4, Supplemental Table S6). The elements with the highest average concentrations (> 1 µg/g) were cadmium, mercury, and selenium in kidney. Chromium was below analytical detection in some muscle samples, lead was below detection in all muscle samples, and arsenic was below detection in a few samples of all tissue types (Supplemental Table S2). The distribution between tissue types varied by element. Cadmium, cobalt, and selenium had the highest concentrations in kidney, followed by intermediate concentrations in liver, and the lowest concentrations in brain and muscle. Mercury also had the highest concentrations in the kidney, while liver concentrations were similar to those in brain and muscle. Nickel and lead had similar concentrations in brain, kidney, and liver but lower concentrations in muscle. Arsenic and chromium had similar or only small differences in concentration between tissues.

The trace element concentrations of Yukon wolverines were generally comparable to values previously reported for wolverines from British Columbia and Nunavut (Table 3). Liver concentrations of cadmium, chromium, cobalt, lead, and mercury were consistently low, < 1 µg/g and often within 1 standard deviation. Nickel and selenium were higher in wolverine liver from British Columbia compared with those from the Yukon. This limited comparison suggests background exposure of those trace elements may be similar in remote regions of the Yukon, British Columbia, and Nunavut.

**Table 3:** Comparison of mean concentrations of trace elements (µg/g dw) in livers of wolverines examined in this study with published data for wolverines from two other regions of Canada

Element	Harding (2004) (British Columbia, n = 11)	Hoekstra et al. (2003a, b) (Nunavut, n = 9–12)	This study (Yukon, n = 25)
Cadmium	0.29 ± 0.11	0.30	0.70 ± 0.41
Chromium	0.05 ± 0.16		0.41 ± 0.47
Cobalt	0.4 ± 0.08		0.06 ± 0.02
Lead	0.18 ± 0.05	< 0.55	0.04 ± 0.03
Mercury	0.18 ± 0.09	0.39	0.18 ± 0.26^a^
Nickel	2.0 ± 1		0.21 ± 0.28
Selenium	6.2 ± 0.8	2.06	2.22 ± 0.44

^a^n = 121

### Arsenic Speciation

**Fig. 5:**
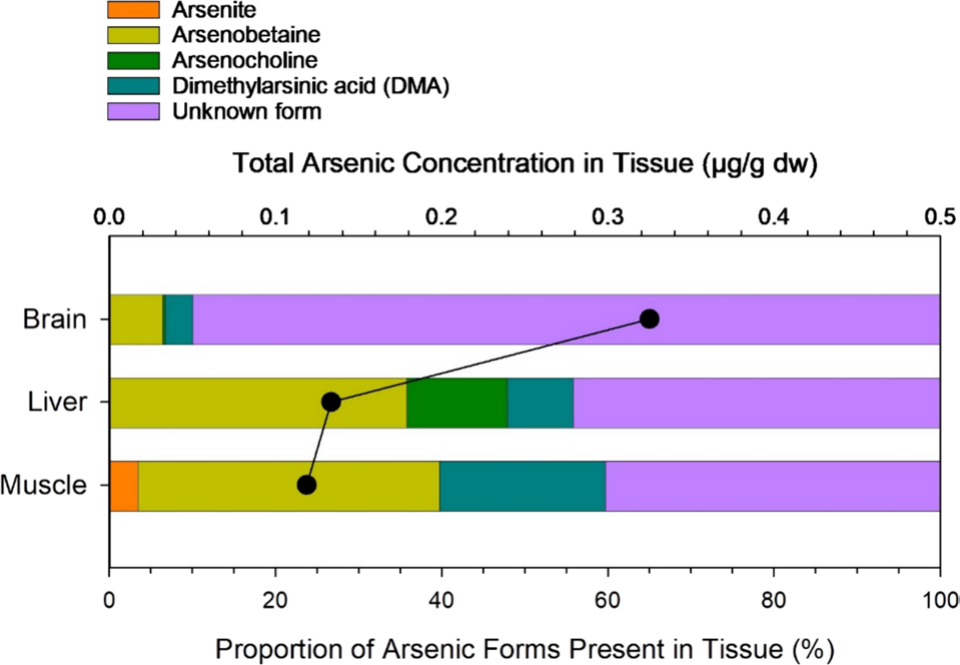
Percent composition of arsenic compounds found in brain, liver, and muscle of Yukon wolverines. Proportions are average values of three animals. The average total arsenic concentration of each tissue type is indicated by the black circles and line

Four arsenic compounds were identified in the brain, liver and muscle of three wolverines from the Yukon (Fig. 5). Arsenobetaine was the dominant compound identified in all three tissues, representing on average 6–36% of total arsenic. Arsenite, arsenocholine and dimethylarsinic acid were also detected, while arsenate and monomethyl arsonic acid were not detected in any of the samples. Interestingly, around half of the total arsenic in muscle and liver was not accounted for by the targeted arsenic compounds and 90% was unaccounted for in the brain. Other arsenic compounds likely also accumulate in wolverine, such as arsenolipids in the brain (Stiboller et al. 2019).

Little information has been reported on arsenic compounds in terrestrial wildlife, and these data (albeit limited) for wolverine contribute to the characterization of arsenic fate in the environment. Diet is the primary exposure pathway of arsenic to wildlife, and the speciation of arsenic in wildlife tissues reflects the composition in the diet as well as internal metabolic processes such as the transformation of inorganic arsenic to methylated arsenic (Popowich et al. 2016; Jamwal et al. 2023). The prevalence of arsenobetaine in terrestrial animals varies; while dominant in wolverine (this study), spruce grouse *Canachites canadensis* and gray jay *Perisoreus canadensis* (Koch et al. 2005), other compounds such as dimethylarsinic acid, arsenate, and arsenite can be more prevalent in other bird species (Koch et al. 2005; Yang et al. 2018) and in hare *Lepus* spp. (Koch et al. 2013). Arsenobetaine is thought to be largely transferred through the food chain, although the origins of this compound remain poorly characterized, particularly in the terrestrial environment (Popowich et al. 2016). Some plants, mushrooms and terrestrial invertebrates contain arsenobetaine (Koch et al. 2013; Moriarty et al. 2009), which may undergo trophic transfer to herbivores and omnivores. The arsenic concentrations of wolverines were low in this study. However, it is possible that animals exposed to point source contamination of inorganic arsenic would have a different tissue composition of arsenic species (Yang et al. 2018).

### Toxicological Risk Assessment

The concentrations of cadmium, lead, mercury, and selenium in wolverines were below tissue burden thresholds associated with a risk of sublethal toxicological effects (Table 4). Those thresholds are based on observed effects (i.e. tissue dysfunction, altered metabolism) in animals with known tissue burdens, and they are commonly presented as ranges for specific types of animals due to variability among species and studies (Ma 2011; Cooke 2011). Cadmium, lead, mercury, and selenium concentrations of wolverines were an order of magnitude below risk thresholds for kidney or liver. Mercury concentrations of wolverines were also below a toxicity risk threshold for the brain, though the values were closer to that threshold than for other tissues we examined. The assessment for wolverine is consistent with a recent risk assessment of mercury exposure to Arctic terrestrial wildlife that found most animals of three species in the circumpolar Arctic had concentrations indicative of no toxicological risk (Dietz et al. 2022). Arctic fox from Iceland were an exception, showing elevated liver mercury in the highest risk category for 9% of animals examined (Dietz et al. 2022). To our knowledge, toxicological risk thresholds for other trace elements (e.g., arsenic, cobalt, nickel) have not been developed and are generally lacking (Rattner et al. 2023).

**Table 4:** Comparison of trace element concentrations in wolverine tissues to risk thresholds associated with potential toxicological effects

Element	Tissue	Observed Concentration (µg/g dw)	Risk Threshold concentration (µg/g dw)	Source
Cadmium	Kidney	0.70–12.5	105–210	Cooke (2011)
Lead	Kidney	0.01–0.31	15–80	Ma (2011)
Mercury	Brain	0.02–1.46	3–5^a^	Dietz et al. (2013)
	Liver	0.01–2.07	14–102^b^	Dietz et al. (2022)
Selenium	Liver	1.57–3.23	20^c^	Ohlendorf and Heinz (2011)

^a^Sub-clinical neurochemical effect threshold

^b^Converted from range of low to high risk thresholds reported on a wet weight basis

^c^Based on waterfowl toxicity data

The brain mercury threshold was used for a risk assessment of sub-clinical effects on neurochemistry, which is a highly sensitive indicator that is not associated with overt toxicological effects. Mercury is a neurotoxin, and the threshold is based on research showing correlations between concentrations of neurotransmitter chemicals and mercury in the brain, which may alter brain function (Basu et al. 2010; Desforges et al. 2021; Dornbos et al. 2013). In contrast with wolverines from this study, which had low brain THg concentrations, river otters from Nova Scotia were found to have brain THg concentrations of up to 18 µg/g dw (Haines et al. 2010). Mercury-associated effects on brain neurochemistry have been observed for river otter (Basu et al. 2005).

Overall, this preliminary assessment suggests the levels of cadmium, lead, mercury, and selenium were not likely to pose a risk of overt toxicological effects on wolverines in this study. Nevertheless, caution is warranted in interpreting risk thresholds because the assessment did not involve measurements of wolverine health. Applying threshold values obtained in laboratory studies to a range of free-living species may not provide accurate indications of health risk. Other factors such as age, diet, and species-specific sensitivity influence metal toxicity (Rattner et al. 2023), and subtle subchronic effects, such as suppression of the immune system, may occur at lower tissue concentrations than the thresholds used in this risk assessment (Desforges et al. 2016).

## Conclusion

We generated the most comprehensive dataset to date on concentrations of mercury and other trace elements in wolverine, including the first measurements for brain, hair, and muscle. Our comprehensive evaluation provides baseline information for future monitoring of contaminant exposure in terrestrial ecosystems of western North America. The choice of tissue for new monitoring efforts may depend on the specific trace element of interest, though kidney generally accumulated the highest concentrations, and hair was a suitable non-lethal indicator for mercury. The observed concentrations in wild wolverines indicated relatively low bioaccumulation of the measured trace elements. Nevertheless, future changes to distant anthropogenic emissions, long-range transport, local industrial developments, and climate change may impact exposure to this terrestrial carnivore at the top of the terrestrial food web in northern ecosystems.

## Supplementary Information

Below is the link to the electronic supplementary material.

Supplementary file1 (DOCX 36 kb)

## Data Availability

The dataset of mercury and trace element concentrations in wolverine tissues is publicly available on Government of Canada Open Data Portal at https://search.open.canada.ca/data/.
