# Supplementary material for: Tissue Distribution and Toxicological Risk Assessment of Mercury and Other Elements in Northern Populations of Wolverine (Gulo gulo)
Source: Arch Environ Contam Toxicol. 2024 Aug 3;87(2):114–26. doi: 10.1007/s00244-024-01081-x (PMC11377595; doi:10.1007/s00244-024-01081-x)
Supplement: Supplementary file 1 — Supplementary file1 (DOCX 36 kb) [file 244_2024_1081_MOESM1_ESM.docx]

**Supplemental Information for:**

**Tissue distribution and toxicological risk assessment of mercury and other elements in northern populations of wolverine (*Gulo gulo*)**

John Chételat*^1^ [ORCID: 0000-0002-9380-7203], Thomas S. Jung^2,3^ [ORCID: 0000-0003-2681-6852], Malik Awan^4^, Steven Baryluk^5^, William Harrower^6^, Piia M. Kukka^2^, Christine McClelland^1^ [ORCID: 0000-0003-2645-0221], Garth Mowat^7,8^, Nicolas Pelletier^9^ [ORCID: 0000-0001-6185-7030], Christine Rodford^1^, Raphaela Stimmelmayr^10,11^ [ORCID: 0000-0001-9384-3901]

^1^ Environment and Climate Change Canada, National Wildlife Research Centre, Ottawa, Ontario, Canada

^2^ Department of Environment, Government of Yukon, Whitehorse, Yukon, Canada

^3^ Department of Renewable Resources, University of Alberta, Edmonton, Alberta, Canada

^4^ Department of Environment, Government of Nunavut, Igloolik, Nunavut, Canada

^5^ Environment and Climate Change, Government of the Northwest Territories, Inuvik, Northwest Territories, Canada

^6^ Department of Forest and Conservation Sciences, University of British Columbia, Vancouver, British Columbia, Canada

^7^ Ministry of Forests, Government of British Columbia, Nelson, British Columbia, Canada

^8^ Department of Earth, Environmental and Geographic Sciences, University of British Columbia, Kelowna, British Columbia, Canada

^9^ Department of Geography and Environmental Studies, Carleton University, Ottawa, Ontario K1S 5B6, Canada

^10^ Department of Wildlife Management, North Slope Borough, Utqiagvik, Alaska, USA

^11^ Institute of Arctic Biology, University of Alaska Fairbanks, Alaska, USA

*Corresponding author. Email: [john.chetelat@ec.gc.ca](mailto:john.chetelat@ec.gc.ca)

Contents

[Table S1. Summary of wolverine tissue sample collections and biological profiles across study regions. 3](#_Toc170224810)

[Table S2. Quality assurance and quality control (QA/QC) data for element analyses of wolverine tissues by ICP-MS. 4](#_Toc170224811)

[Table S3. Quality assurance and quality control (QA/QC) data for arsenic speciation measurement of wolverine tissues. 5](#_Toc170224812)

[Table S4. Predictive linear regression equations to estimate brain, kidney, and liver THg concentrations of wolverine as a function of muscle and hair THg concentration. 6](#_Toc170224813)

[Table S5. Comparison of conversion factors (based on linear regression equations) among mustelid species to estimate brain, kidney, and liver THg concentrations as a function of muscle and hair THg concentration. 7](#_Toc170224814)

[Table S6. Mean (± standard deviation) and range of trace element concentrations in muscle, liver, kidney and brain of 25 wolverine from the Yukon. 8](#_Toc170224815)

Table S1. Summary of wolverine tissue sample collections and biological profiles across study regions.

| **Region** | **Number of Wolverines** | **Males: Females** | **Juveniles: Adults** | **Tissue Types** | **Years of Collection** |
| --- | --- | --- | --- | --- | --- |
| Alaska | 17 | 13:4 | 4:13 | kidney, liver, muscle | 2014 - 2018 |
| British Columbia | 22 | 9:13 | n.a. | hair | 2009 - 2016 |
| Northwest Territories | 99 | 62:32 | 39:58 | Brain, hair, liver, kidney, muscle | 2006 - 2018 |
| Nunavut | 44 | 35:9 | 0:45 | Hair, muscle | 2010 - 2014 |
| Yukon | 322 | 201:120 | 103:95 | Brain, hair, liver, kidney, muscle | 2005 - 2018 |

n.a. = not available

Table S2. Quality assurance and quality control (QA/QC) data for element analyses of wolverine tissues by ICP-MS. Results are summarized for analytical duplicates (mean and range of the relative percent difference [RPD]), detection limits (DL), percent of sample results below analytical detection, and recoveries of certified reference materials (CRMs).

| **Element** | **Duplicate RPD (%)** | | **Detection Limit (µg/g)** | **Results**  **< DL (%)** | **CRM Recovery (%)** | | **CRM Precision**  **(% RSD)** | **CRMs Analyzed** |
| --- | --- | --- | --- | --- | --- | --- | --- | --- |
|  | **Mean (Range)** | **N** |  |  | **Mean (Range)** | **N** |  |  |
| As | 10 (2-25) | 8 | <0.005 | 12 | 98 (89-110) | 18 | 0.7-2.1 | A,B,C |
| Cd | 8 (0-40) | 11 | <0.002 | 1 | 94 (86-102) | 18 | 1.0-2.5 | A,B,C |
| Co | 3 (0-7) | 12 | <0.005 | 0 | 98 (93-103) | 7 | 1.2 | B |
| Cr | 39 (4-173) | 11 | <0.05 | 10 | 95 (80-112) | 11 | 2.7-4.7 | A,C |
| Ni | 22 (0-62) | 11 | <0.02 | 0 | 99 (87-116) | 11 | 2.9 | A,C |
| Pb | 11 (3-24) | 9^a^ | <0.005 | 25 | 107 (92-117) | 18 | 0.9-2.9 | A,B,C |
| Se | 6 (0-16) | 12 | <0.005 | 0 | 99 (86-107) | 18 | 0.5-1.2 | A,B,C |

^a^ One duplicate measurement of brain sample was excluded due to contamination from Pb ammunition particles

**CRMs:**

A – NRC DORM-4 Fish

B – NRC DOLT-5 Dogfish Liver

C – NIST Mussel Tissue 2976

Table S3. Quality assurance and quality control (QA/QC) data for arsenic speciation measurement of wolverine tissues. Results are summarized for analytical duplicates mean of the relative percent difference [RPD]), detection limits (DL), percent of sample results below analytical detection, and recoveries of certified reference materials (CRMs). Concentrations were determined on a wet weight basis.

| **Arsenic Speciation** | **Duplicate RPD (%)** | | **Detection Limit (µg/g)** | **Results**  **< DL (%)** | **CRM Recovery (%)** | | **Recovery Matrix** |
| --- | --- | --- | --- | --- | --- | --- | --- |
|  | **Mean** | **N** |  |  | **Mean (Range)** | **N** |  |
| Arsenate | ND | 2 | <0.0050 | 100 | 101 (83-117) | 4 | LCS, RM |
| Arsenite | 2 | 2 | <0.0010 | 89 | 86 (80-93) | 4 | LCS, RM |
| Arsenobetaine | 2 | 2 | <0.0010 | 11 | 97 (96-97) | 2 | LCS |
| Arsenocholine | 3 | 2 | <0.0010 | 56 | 97 (95-99) | 2 | LCS |
| DMA | 4 | 2 | <0.0010 | 22 | 92 (82-104) | 4 | LCS, RM |
| MMA | 12 | 2 | <0.0010 | 100 | 97 (95-100) | 4 | LCS, RM |

LCS = laboratory control sample

RM = reference material

Table S4. Predictive linear regression equations to estimate brain, kidney, and liver THg concentrations of wolverine as a function of muscle and hair THg concentration. The equations were calculated using ordinary least-squares regression with the y-intercept forced to zero, data were square-root transformed, and the coefficients and standard errors are presented as back-transformed values.

| **Predictor Variable (X)** | **Response Variable (Y)** | **Beta Regression Coefficient** | **Standard Error** | **Adj R^2^** | **Sample Size** |
| --- | --- | --- | --- | --- | --- |
| Muscle | Brain | 0.28 | 0.01 | 0.98 | 47 |
| Muscle | Kidney | 3.23 | 0.10 | 0.96 | 195 |
| Muscle | Liver | 0.78 | 0.03 | 0.96 | 143 |
| Hair | Brain | 0.11 | 0.04 | 0.67 | 14 |
| Hair | Kidney | 1.03 | 0.12 | 0.80 | 79 |
| Hair | Liver | 0.27 | 0.04 | 0.78 | 54 |
| Hair | Muscle | 0.27 | 0.02 | 0.84 | 107 |

Table S5. Comparison of conversion factors (based on linear regression equations) among mustelid species to estimate brain, kidney, and liver THg concentrations as a function of muscle and hair THg concentration. Conversion factors for river otter and mink were obtained from Eccles et al. (2017).

| **Predictor Variable (X)** | **Response Variable (Y)** | **Beta Regression Coefficient ± SE** | | |
| --- | --- | --- | --- | --- |
|  |  | **Wolverine** | **Mink** | **River Otter** |
| Muscle | Brain | 0.28 ± 0.01 | 0.43 ± 0.10 | 0.39 ± 0.03 |
| Muscle | Kidney | 3.23 ± 0.10 | 0.77 ± 0.05 | 1.94 ± 0.11 |
| Muscle | Liver | 0.78 ± 0.03 | 1.10 | 2.02 ± 0.14 |
| Hair | Brain | 0.11 ± 0.04 | 0.13 ± 0.02 | 0.15 ± 0.01 |
| Hair | Kidney | 1.03 ± 0.12 | 0.64 ± 0.05 | 0.62 ± 0.05 |
| Hair | Liver | 0.27 ± 0.04 | 0.46 ± 0.03 | 0.70 ± 0.03 |
| Hair | Muscle | 0.27 ± 0.02 | 0.30 ± 0.04 | 0.46 ± 0.06 |

Table S6. Mean (± standard deviation) and range of trace element concentrations in muscle, liver, kidney and brain of 25 wolverine from the Yukon. Detection limit values were substituted for non-detects in the calculation of means and standard deviations.

| **Element** | **Muscle**  **(µg/g dw)** | **Liver**  **(µg/g dw)** | **Kidney**  **(µg/g dw)** | **Brain**  **(µg/g dw)** |
| --- | --- | --- | --- | --- |
| Arsenic | 0.041 ± 0.058  (<0.005 – 0.231) | 0.034 ± 0.049  (<0.005 – 0.202) | 0.039 ± 0.055  (<0.005 – 0.183) | 0.109 ± 0.256  (<0.005 – 1.05) |
| Cadmium | 0.014 ± 0.020  (0.002 – 0.105) | 0.697 ± 0.413  (0.098 – 1.63) | 4.18 ± 3.02  (0.704 – 12.5) | 0.010 ± 0.009  (<0.002 – 0.038) |
| Chromium | 0.35 ± 1.26  (<0.05 – 6.41) | 0.41 ± 0.47  (0.12 – 2.33) | 0.48 ± 0.55  (0.15 – 3.04) | 0.26 ± 0.32  (0.05 – 1.72) |
| Cobalt | 0.021 ± 0.006  (0.013 – 0.036) | 0.062 ± 0.021  (0.032 – 0.110) | 0.158 ± 0.041  (0.098 – 0.278) | 0.025 ± 0.007  (0.015 – 0.055) |
| Lead | <0.005 | 0.038 ± 0.030  (0.007 – 0.137) | 0.051 ± 0.065  (0.009 – 0.305) | 0.050 ± 0.151  (0.006 – 0.707) |
| Nickel | 0.07 ± 0.06  (0.02 – 0.25) | 0.21 ± 0.28  (0.05 – 1.32) | 0.13 ± 0.05  (0.04 – 0.27) | 0.19 ± 0.21  (0.05 – 1.09) |
| Selenium | 0.617 ± 0.159  (0.422 – 1.04) | 2.22 ± 0.44  (1.57– 3.23) | 5.99 ± 0.85  (4.31 – 7.58) | 1.02 ± 0.21  (0.727 – 1.62) |
